# The Potential Cytotoxic Activity Enhancement of α-Mangostin in Chitosan-Kappa Carrageenan-Loaded Nanoparticle against MCF-7 Cell Line

**DOI:** 10.3390/polym13111681

**Published:** 2021-05-21

**Authors:** Nasrul Wathoni, Lisna Meylina, Agus Rusdin, Ahmed Fouad Abdelwahab Mohammed, Dorandani Tirtamie, Yedi Herdiana, Keiichi Motoyama, Camelia Panatarani, I Made Joni, Ronny Lesmana, Muchtaridi Muchtaridi

**Affiliations:** 1 Department of Pharmaceutics and Pharmaceutical Technology, Faculty of Pharmacy, Universitas Padjadjaran, Sumedang 45363, Indonesia; lisna@farmasi.unmul.ac.id (L.M.); agusrusdin@gmail.com (A.R.); farmasetikers@gmail.com (D.T.); y.herdiana@unpad.ac.id (Y.H.); 2 Department of Pharmaceutics and Pharmaceutical Technology, Faculty of Pharmacy, Universitas Mulawarman, Samarinda 75119, Indonesia; 3 Department of Pharmacy, Faculty of Sports and Health, Universitas Negeri Gorontalo, Gorontalo 96128, Indonesia; 4 Department of Pharmaceutics, Faculty of Pharmacy, Minia University, Minia 61519, Egypt; ahmed.mohamed1@minia.edu.eg; 5 Graduate School of Pharmaceutical Sciences, Kumamoto University, Kumamoto 862-0973, Japan; motoyama@kumamoto-u.ac.jp; 6 Department of Physics, Faculty of Mathematics and Natural Sciences, Universitas Padjadjaran, Sumedang 45363, Indonesia; c.panatarani@unpad.ac.id (C.P.); imadejoni@phys.unpad.ac.id (I.M.J.); 7 Functional Nano Powder University Center of Excellence, Universitas Padjadjaran, Sumedang 45363, Indonesia; 8 Department of Anatomy, Physiology and Biology Cell, Faculty of Medicine, Universitas Padjadjaran, Sumedang 45363, Indonesia; ronny@unpad.ac.id; 9 Department of Pharmaceutical Analysis and Medicinal Chemistry, Faculty of Pharmacy, Universitas Padjadjaran, Sumedang 45363, Indonesia; muchtaridi@unpad.ac.id

**Keywords:** chitosan, cytotoxic, kappa carrageenan, α-mangostin, polymeric nanoparticle

## Abstract

α-mangostin (αM), a xanthone derivative compound isolated from the extract of mangosteen pericarp (*Garcinia mangostana* L), has potential anticancer properties for breast cancer. However, it has poor solubility in water and low selectivity towards cancer cells. The polymeric nanoparticle formulation approach can be used to overcome these problems. In this study, a chitosan biopolymer-based αM polymeric nanoparticle formulation was encapsulated using kappa carrageenan (αM-Ch/Cr) as a novel carrier for breast cancer therapy and evaluated for their physicochemical properties, drug release profile, and in vitro cytotoxicity against breast cancer cells (MCF-7). Polymeric nanoparticles formulated with varying concentrations of kappa carrageenan were successfully prepared by ionic gelation and spray pyrolysis techniques. αM-Ch/Cr nanoparticles formed perfectly round particles with a size of 200–400 nm and entrapment efficiency ≥ 98%. In vitro release studies confirmed that αM-Ch/Cr nanoparticles had a sustained release system profile. Interestingly, the formulation of polymeric nanoparticles significantly (*p* < 0.05) increased the cytotoxicity of αM against MCF-7 cell with IC50 value of 4.7 μg/mL compared to the non-nanoparticle with IC50 of 8.2 μg/mL. These results indicate that αM-Ch/Cr nanoparticles have the potential to improve the physicochemical properties and cytotoxicity effects of αM compounds as breast cancer therapy agents.

## 1. Introduction

Breast cancer is the second most prevalent cancer after lung cancer, accounting for 11.6% of all cancer cases (2,088,849 cases) and 6.6% of all cancer-related deaths (626,679 deaths) in 2018 [1]. Breast cancer is currently treated with local treatment (surgery and radiation) and systemic treatment (chemotherapy, hormone therapy, targeted drug therapy, and immunotherapy) [2]. However, these therapeutic methods have many side effects. Besides, the efficacy of chemotherapeutic agents is also reduced by the presence of cancer cell resistance (multi-drug resistance mechanism) [3,4].

Nowadays, natural ingredients are mostly used for alternative cancer treatment. α-mangostin (αM), a secondary metabolite isolated from pericarp of mangosteen (*Garcinia mangostana* Linn), had been reported to have a potent cytotoxic effect by induction of apoptosis in various cancer cell lines [5]. αM induced apoptosis in breast cancer cells through the inhibition of fatty acid synthase via the signaling pathway of human epidermal growth factor receptor-2 (HER2)/phosphatidylinositide 3-kinase (PI3K)/Akt and mitogen-activated protein kinase (MAPK). Despite its potent cytotoxic effect and anticancer activity, αM has limitations in physicochemical properties that need to be considered, including low solubility and its non-selectivity to cancer cells [6,7]. These properties reduce its effectiveness in treatment of cancer.

In a polymeric nanoparticle drug delivery system, nanoparticles are utilized as drug carriers. Some studies suggest that the use of polymeric nanoparticles as a drug delivery system can increase drug solubility, provide a controlled drug release, and accumulate in cancer tissues through enhanced permeability and retention effect (EPR), allowing for more specific targeting [8,9,10,11]. Chitosan, a biopolymer obtained by alkaline deacetylation of chitin, has properties as a promising material for nanoparticle drug delivery [12,13,14]. The mucoadhesive properties of chitosan can increase drug penetration into cancer cells and suppress drug efflux reactions to maximize the level of drug entering the cells. In addition, chitosan nanoparticles can inhibit cell migration activity in MDA-MB-231 [15], and improve anticancer activities in HeLa [16], HUVE [17], and HCC cells [18]. The activities of chitosan in killing cancer cells are known to work through the mechanism of induction of apoptosis through activation of caspases 3, 8, 9; modulation ratio of Bax: Bcl-2; induce DNA damage [19,20] and the characteristics of chitosan undergo protonation in acidic environments and provide useful release at low pH as in tumor microenvironment that has acidic pH [21]. However, in oral administration, this activity can decrease due to the protonation of the amine group of chitosan [22,23,24].

Enteric-coated nanoparticles are one of the drug delivery systems in oral administration that can protect the reaction of degradation and control the release of encapsulated drugs in specific parts of the gastrointestinal tract. Kappa carrageenan is often used as a coating material because of its low sensitivity to pH and ionic strength [25]. Owing to its negative charge, kappa carrageenan is commonly used in combination with cationic or polymeric cationic polymerized electrolytes as a microcapsule shell. Kappa carrageenan is also used together with chitosan in the manufacture of microcapsules containing glucose oxidase for controlled release for oral administration [26] and nanoparticles containing recombinant human erythropoietin [27], ciprofloxacin [28], and SiO_2_–NH_2_ [29].

Following a review of the literature, there are no studies reported using chitosan polymeric nanoparticles loaded with kappa carrageenan as a drug delivery system for αM. Therefore, the main objective of this study was to prepare and characterize αM nanoparticles, for improving the solubility and anticancer activity of αM mediated by nanoparticle drug delivery system.

**Figure 1:**
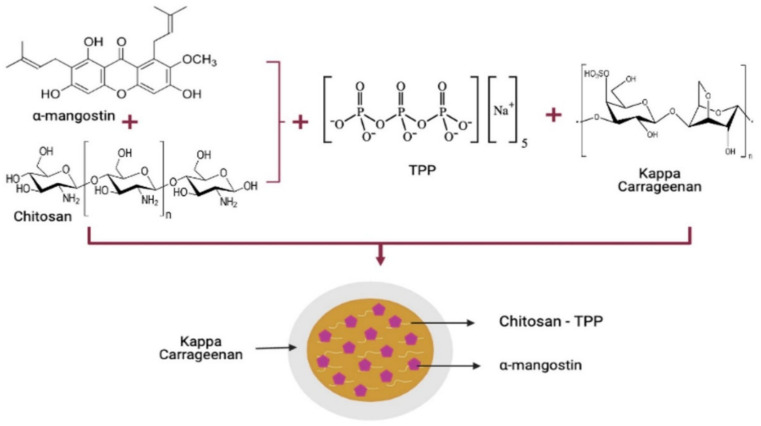
Synthesis of αM chitosan-kappa carrageenan nanoparticles.

Therefore, the main objective of this study was to prepare the nanoparticle drug delivery system for the oral route of administration. Chitosan, the main carrier for providing solubility and penetration improvement, is known to have instability at gastric pH, therefore, it is was added with carrageenan as an encapsulator for protecting chitosan protonation from the gastric fluid (Figure 1). In addition, the physicochemical characterization of the nanoparticle system will characterize and evaluate the release profile of nanoparticles in fluids with different pHs (1.2 and 7.4) for representing gastrointestinal tract condition especially in stomach (pH 1.2) and ileum (pH 7.4). Furthermore, the cytotoxic assay was studied on breast cancer (MCF-7 cell lines) to evaluate the αM polymeric nanoparticle cytotoxic effect.

## 2. Materials and Methods

### 2.1. Material

α-mangostin (αM) was purchased from Chengdu Biopurify Phytochemicals (Chengdu, Sichuan, China). Chitosan was isolated with a purity of 70% (MW: 1526.5 g/mol, DD: 81.38%). Sodium tripolyphosphate and kappa carrageenan (MW: 788.7 g/mol) were purchased from Kappa Carrageenan Nusantara (Bekasi, West Java, Indonesia), ethanol from Kristata (Bandung, West Java, Indonesia), and acetic acid solvent from Brataco (Bandung, West Java, Indonesia). MCF-7 human breast cancer cell line was purchased from the American Type Culture Collection (Manassas, VA, USA).

### 2.2. Preparation of αM Chitosan Nanoparticles

The αM chitosan (αM-Ch) nanoparticles was prepared by the ionic gelation method. Briefly, 20 mg α-mangostin was diluted in 20 mL ethanol. Then, it was mixed with 0.1% *w/v* (200 mg/200 mL) of chitosan solution prepared in 1% acetic acid. Next, 0.1% *w/v* (40 mg/40 mL) sodium tripolyphosphate solution was added drop wise under constant magnetic stirring. Then, the system was ultrasonicated for 30 min to reduce the droplet size in the suspension system [30].

### 2.3. Preparation of αM Chitosan-Loaded Kappa Carrageenan Nanoparticles

The αM chitosan-loaded kappa carrageenan (αM-Ch/Cr) nanoparticles were prepared by the solvent evaporation method. For the coating process, αM-Ch was dropped into varying concentrations of kappa carrageenan (F1: 25 mg/25 mL, F2: 45 mg/45 mL, and F3: 85 mg/85 mL) (Table 1). The solution mixture was conducted via microvolume flow titration method, and then, dried nanoparticles were obtained by drying the suspension solutions using spray pyrolysis at a temperature of 80–100 °C and at airflow of 5 L/min [30].

### 2.4. Scanning Electron Microscopy (SEM)

Scanning electron microscopy (Model SU3500 SEM; Hitachi, Tokyo, Japan) was used to examine the morphology of αM-Ch/Cr nanoparticles. Briefly, αM-Ch/Cr nanoparticles were placed into the stub and were coated with platinum (30 s, 10 mA). The photomicrographs of αM-Ch/Cr nanoparticles were observed at a 10 kV with differential magnifications (×10,000—50,000). Data were analyzed using ImageJ software (Madison, WI, USA) and Origin software (version 8.5, Northampton, MA, USA) to obtain the average particle size and distribution [30].

### 2.5. Entrapment Efficiency and Drug Loading

The αM-Ch/Cr entrapment efficiency and drug loading of nanoparticles were estimated by UV–VIS spectroscopy. Briefly, 25 mg sample of nanoparticles were added to ethyl acetate and the solution was centrifuged (3000 rpm, 10 min). Next, the supernatant was collected and the absorption at 245 nm was measured with an UV–visible spectrophotometer using a standard curve to obtain the amount of free αM. Then, the sediment was resuspended in an appropriate volume of ethanol to determine the drugs that were encapsulated to find the total amount of αM. To obtain the standard curve, different concentrations (2–12 µg/mL) of αM with serial dilution method were prepared and measured at 245 nm. The entrapment efficiency and drug loading of αM in αM-Ch/Cr nanoparticles were determined by the following Equations (1) and (2) [30].

(1) $$\mathrm{Entrapment}\;\mathrm{efficiency}\left(\%\right)=\frac{\mathrm{weight}\;\mathrm{of}\;\mathrm{the}\;\alpha M\;\mathrm{in}\;\mathrm{nanoparticle}}{\mathrm{weight}\;\mathrm{of}\;\alpha M\;\mathrm{used}}\times 100\%$$

(2) $$\mathrm{Drug}\;\mathrm{loading}\;\left(\%\right)=\frac{\mathrm{weight}\;\mathrm{of}\;\mathrm{the}\;\alpha M\;\mathrm{in}\;\mathrm{nanoparticle}\;}{\mathrm{weight}\;\mathrm{of}\;\alpha M\;\mathrm{nanoparticle}}\times 100\%.$$

### 2.6. Fourier-Transform Infrared Spectroscopy (FTIR)

The interaction between αM, chitosan (Ch), sodium tripolyphosphate (Tpp), kappa carrageenan (Cr), and αM-Ch/Cr nanoparticles was characterized using a Fourier-transform infrared spectrophotometer (Model IR Prestige-21; Shimadzu, Kyoto, Japan) with vacuum pressure (60 kN within 15 min) and measured at 4000−400 cm^−1^ [30,31].

### 2.7. X-ray Diffraction (XRD)

The molecular arrangements of the αM-Ch/Cr nanoparticle system were observed using x-ray diffraction (X-pert MPD diffractometer type, Rigaku International, Tokyo, Japan). The XRD patterns were collected over the angular range (2θ) of 5–60°, with the generator set to 30 mA and 40 kV. The same condition was used for raw material analysis [30,32].

### 2.8. Differential Scanning Calorimetry (DSC)

The thermal behavior of all raw materials and αM-Ch/Cr nanoparticles was studied by differential scanning calorimetry (DSC-60; Mettler Toledo, Barcelona, Spain) and analyzed using TA-60WS software (Mettler Toledo, Barcelona, Spain). All measurements were performed at temperatures from 30 to 300 °C, with a heating rate of 20 °C/min under nitrogen flow [30,33].

### 2.9. Saturation Solubility Study

The αM-Ch/Cr nanoparticles were put into 5 mL distilled water pH 7.4, then constantly stirred for approximately 24 h. The sample was then filtered and diluted at a ratio of 1:3. Then, the αM level was measured using a UV–VIS spectrophotometer at a wavelength of 245 nm [34].

### 2.10. In Vitro Drug Release

The αM-Ch/Cr nanoparticle release profiles at pH 7.4 in phosphate buffered saline (PBS) and at a pH of 1.2 in a hydrochloric acid (HCl) solution were studied. Typically, 25 mg of αM-Ch/Cr nanoparticles were suspended in PBS (pH 7.4) and in HCl solution (pH 1.2) in separate containers. Then, a 5 mL sampling was collected at each time interval of 5, 10, 15, 30, 45, and 60 min followed by the addition of fresh media. Furthermore, measurements were taken using a spectrophotometer instrument at a wavelength of 245 nm and the percent of drug released at each time interval was calculated [35].

### 2.11. In Vitro Cytotoxicity

The cell cytotoxicity of αM, Ch-TPP, αM-Ch, and αM-Ch/Cr on MCF-7 cells were determined using MTT assay. Briefly, cells were seeded in a 96-well plate at a seeding density of 5 × 10^3^ cells/well and allowed to attach for 24 h. The following day, media was removed and replaced with a fresh one containing the different samples at varying concentrations (2:12 µg/mL). Cells were incubated for 24 h. Media were removed and replaced with MTT solution at a concentration of 0.5 mg/mL and incubated for an additional 2–4 h at 37 °C. Next, 100 µL SDS in 0.01% HCl was added and incubated in a dark place at room temperature overnight. The absorbance of the formed formazan was measured at 450 nm using ELISA Reader (Epoch™ Microplate Spectrophotometer, BioTek, Winooski, VT, USA). The cell viability was presented as a percentage of the control cells not exposed to the samples, as shown in Equation (3), and median inhibitory concentrations (IC_50_) were calculated from the dose–response curves [36]:

(3) $$Cell\;viability\;\left(\%\right)=\frac{absorbance\;at\;450\;\mathrm{nm}\;of\;treated\;sample}{absorbance\;at\;450\;\mathrm{nm}\;of\;control\;sample}\times 100.$$

### 2.12. Statistical Analysis

Statistical analysis was completed using the Student’s *t*-test. *p*-Values less than 0.05 were considered to be significant. Data are presented as mean ± SD, *n* = 3 independent treatments.

## 3. Results

### 3.1. Characterization of Nanoparticles

#### 3.1.1. SEM, Particle Size, Entrapment Efficiency, and Drug Loading

**Figure 2:**
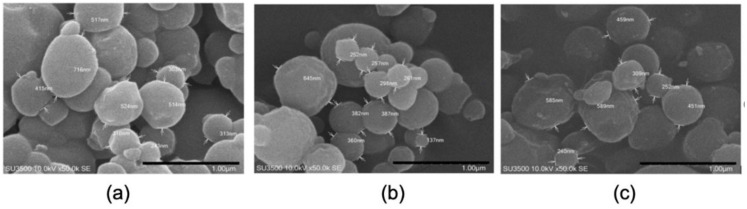
SEM images of (**a**) F1, (**b**) F2, and (**c**) F3.

**Figure 3:**
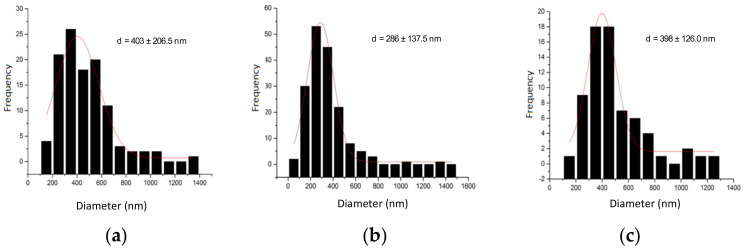
SEM size distribution analysis of (**a**) F1, (**b**) F2, and (**c**) F3.

The SEM images of the αM-Ch/Cr nanoparticles are shown in Figure 2. Upon morphological characterization of the three αM-Ch/Cr nanoparticle formulas, it was observed that all formulations formed spherical shapes. The particle size, entrapment efficiency, and drug loading of the polymeric nanoparticles are shown in Figure 3 and Table 2. In this study formula (2) was selected for further characterization and cytotoxic testing against MCF-7 breast cancer cells.

#### 3.1.2. FTIR Analysis

**Figure 4:**
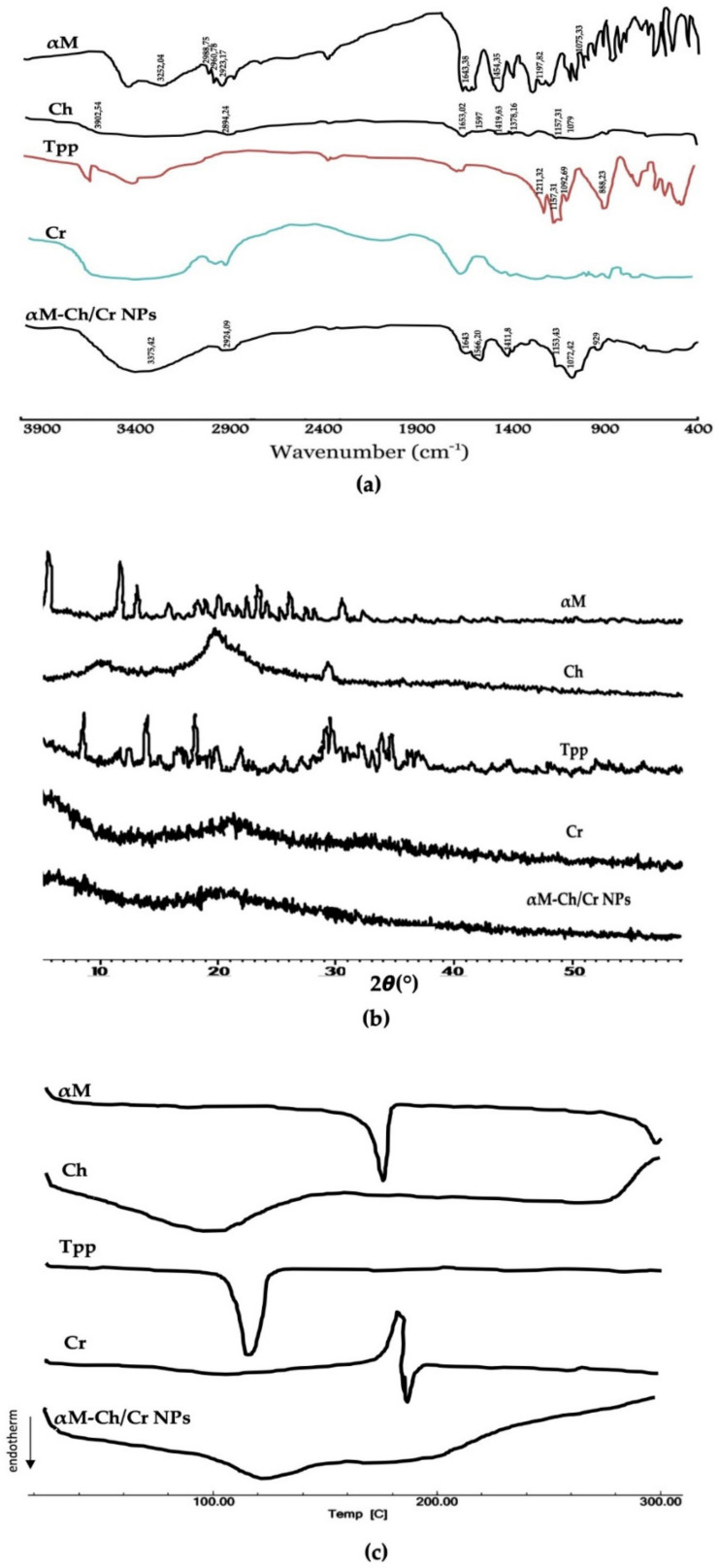
(**a**) FTIR spectra, (**b**) X-ray patterns, and (**c**) DSC thermograms of α-mangostin, chitosan, carrageenan, and αM-Ch/Cr nanoparticles.

The FTIR spectrum of αM, Ch, Tpp, Cr, and αM-Ch/Cr nanoparticles are shown in Figure 4a and the data wavenumber of αM, Ch, Tpp, and Cr are shown in Table 3. The spectrum of αM-Ch/Cr nanoparticle showed the overlapping response of O–H and N–H at 2400 cm^−1^, C–H stretch at 2900 cm^−1^, N–H bend at 1600 cm^−1^ from αM and Ch, 3,6-anhydrogalactose at 929 cm^−1^, and C–O stretch at 1076 cm^−1^ from Cr.

#### 3.1.3. XRD Analysis

The results of the XRD analysis can be seen in Figure 4b. Crystalline patterns from diffractogram data are shown by αM and TPP with sharp diffractogram peaks from 2θ angles of 7–32°, semi-crystalline patterns are shown by chitosan at 2θ angle of 19.72° and amorphous patterns are shown by kappa carrageenan. The XRD results of αM-Ch/Cr nanoparticles demonstrated an amorphous pattern.

#### 3.1.4. DSC Analysis

The results of the DSC analysis are illustrated in Figure 4c. The DSC thermograms for αM and TPP show the peaks of the endothermic phase at 178 and 120.06 °C. Ch appears to provide a wide response signal that peaks around 100 °C. The αM-Ch/Cr nanoparticles show patterns following the glass transition pattern, and the endothermic or exothermic peaks of crystalline component components such as αM and TPP are no longer visible.

### 3.2. In Vitro Studies

#### 3.2.1. Saturation Solubility Study

**Figure 5:**
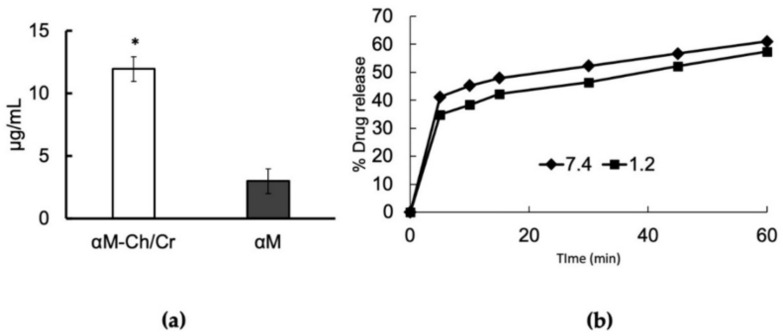
(**a**) Saturation solubility study of αM and αM-Ch/Cr nanoparticle and (**b**) release profiles at (■) pH 1.2 and (♦) pH 7.4 of αM from αM-Ch/Cr nanoparticle. Data represent mean ± SD. * *p* < 0.05, compared to αM (*p* < 0.05).

Saturation solubility study results are shown in Figure 5a. In this study, an increase in the value of αM solubility was four times that of the αM-Ch/Cr nanoparticles (2.98–11.94 µg/mL).

#### 3.2.2. In Vitro Release

The αM-Ch/Cr nanoparticle in vitro release was evaluated in PBS (pH 7.4) and hydrochloric acid solution (pH 1.2) over a period of 60 min. The release profile of αM-loaded nanoparticles exhibited an initial burst release of 34% and 42% for pH 1.2 and 7.0, respectively, over the first 5 min and then followed by a slow and sustained release over a period of 60 min (Figure 5b).

#### 3.2.3. In Vitro Cytotoxicity

**Figure 6:**
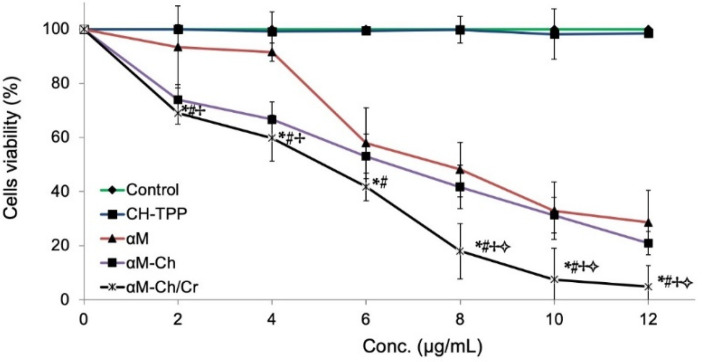
In vitro cytotoxicity of α-mangostin, αM-Ch, and αM-Ch/Cr in MCF-7 cells based on the MTT assay. Data represent mean ±SD. * *p* < 0.05, compared to the control. # *p* < 0.05, compared to the CH-TPP. ✢ *p* < 0.05, compared to the αM. **✧** *p* < 0.05, compared to the αM-Ch.

For the in vitro cytotoxicity study, the cytotoxicity of Ch-TPP, αM, αM-Ch, and αM-Ch/Cr nanoparticles were investigated after incubation with MCF-7 cells for 24 h (Figure 6). No cytotoxic effect on cells was observed for Ch-TPP. There was a significant difference between the cytotoxicity of αM, αM-Ch, and αM-Ch/Cr nanoparticles. The IC_50_ of αM, αM-Ch, and αM-Ch/Cr was 8.2, 6.7, and 4.7 μg/mL, respectively.

## 4. Discussion

This study aims to develop an orally administered nanoparticle polymeric system of α-mangostin as a chemotherapy drug for breast cancer. The polymeric nanoparticles, using chitosan as a carrier and kappa carrageenan as a coating agent, were prepared using the ionic gelation method and spray pyrolysis.

Scanning electron microscopic imaging of the three αM-Ch/Cr nanoparticle formulas revealed spherical nanoparticles. From the particle size, entrapment efficiency, and drug loading of polymeric nanoparticles (Table 2), it can be seen in each formula that there is no real correlation between variations of kappa carrageenan concentration and particle size or entrapment efficiency. Generally, particle size is strongly influenced by the concentration of material in each system, wherein the particle size will be larger for formula that contains a greater amount of ingredients [41]. However, there are certain conditions where there is no relationship between the amount of ingredients and the particle size. This could be due to the unevenness of the frequency of exposure received by the nanoparticle system at the time of manufacture to affect its size, as well as factors such as stirring speed, duration of stirring, duration of ultrasonication, temperature, and system pH [42]. In addition, this pattern can also be caused by the physicochemical properties of some polymers that have a strong attractive interaction with other particles due to the large surface free energy or high affinity for water molecules, which can cause fluid, osmotic, and thermodynamic imbalances that cause agglomeration of particles [43,44], so that the particle size obtained does not provide a clear pattern of relationship between its effect on the concentration of the material. Furthermore, the kappa carrageenan concentration in the formula affects drug loading, where the higher kappa carrageenan concentration will produce a smaller drug loading.

The size of the particles for cancer therapy drug delivery systems plays an important role, it can affect cellular uptake through the process of endocytosis and determines its fate during systemic circulation. In particular, particles with sizes ranging from 100 to 200 nm have the advantage of targeted delivery in cancer compared to those of larger sizes [45]. This is an additional justification for choosing formula 2 for further evaluation to determine its characteristics and cytotoxicity.

The αM-Ch/Cr nanoparticle shows similar transmission peaks to those of Cr, with an absorption band at the wave number 2400 cm^−1^ indicating the overlapping response of the hydroxy group and amine (OH and NH), aliphatic hydrocarbons (CH) 2900 cm^−1^, NH bending from the primary amine at 1600 cm^−1^, interactions between ammonium ions and phosphate 1500 cm^−1^; carrageenan-specific functional groups were also observed at a peak of 929 cm^−1^ indicating the presence of 3.6 anhydrogalactose groups; and glycosidic bonds were observed in areas around 1076 cm^−1^. The data indicate that the α-mangostin was successfully loaded into nanoparticles system.

The diffractogram data for αM-Ch/Cr nanoparticle demonstrates the amorphous pattern, or the transformation of crystalline or semicrystalline phases from the material component to amorphous is seen. The crystalline lattice of αM is no longer visible, which indicates that αM has been dispersed evenly in the nanoparticle matrix and encapsulated in the nanoreservoir system. In addition, the semi-crystalline change from chitosan is assumed to be due to the termination of the amine and hydroxy groups, which results in the formation of amorphous complexes with polymer encapsulators. This is also supported by DSC thermograms that shows the existence of endothermic phase peaks at 178.00 and 120.06 °C as the melting points of αM and TPP. The pattern also illustrates that both materials are crystalline. Although Ch demonstrated a glass transition pattern, which indicates an amorphous shape, DSC thermograms showed an endothermic peak around 100 °C. This is the response from the H_2_O evaporation phase, which is bound in a heat-induced chitosan molecule [46]. The αM-Ch/Cr nanoparticles also show patterns following the glass transition pattern, and the endothermic or exothermic peaks of crystalline components such as αM and TPP are no longer visible. Based on the correlation with XRD as seen in previous tests, it was proved that αM-Ch/Cr nanoparticles were molecularly dispersing αM in the nanoparticle matrix system and encapsulated in the nanoreservoir system.

The αM-Ch/Cr nanoparticles showed a 4-fold increase in the solubility value, likely due to the contribution of hydrophilic polymers used in the formula and also the nature of nanoparticles, which tend to increase the total surface area of the substance in contact with the solvent medium, resulting in a significant increase in solubility [47,48,49].

The in vitro release profile of αM from αM-Ch/Cr nanoparticles provides an extended-release pattern, due to the role of kappa-carrageenan, which is known to be able to control the drug release through the swelling mechanism. The dissolution process of nanoparticles occurs when they come in contact with the solvent medium forming cavities that act as the entry point for the solvent into the system and then dissolve the active substance. These then partition slowly out of the nanoreservoir system and provide an extended-release profile [50]. Based on the drug release curve, a burst release profile of 34% and 42% for pH 1.2 and 7.0, respectively, can be seen over the first 5 min and then followed by a controlled release over a period of 1 h. Burst release profiles are assumed to be due to the presence of αM on the surface of the nanoparticles, which are immediately released when the nanoparticles are in contact with the solvent medium [51]. In addition, there was no significant difference between the release profiles in medium with two different pH conditions. This is due to the nature of carrageenan, which is not a pH-sensitive polymer but rather a thermal-sensitive polymer [25]. In fact, it can be seen that the value of α-mangostin released at pH 1.2 tends to be lower than at pH 7.0 supporting another point of view about the ability of carrageenan in protecting the main carrier system of chitosan from protonation reaction mediated by gastric fluid.

In vitro cytotoxicity evaluation showed that the cell growth treated with the same anticancer concentration but in different formulations shows a different inhibitory response to the growth of cancer cells. When MCF-7 cells were treated with a dose of 2 and 4 µg/mL of αM in various formulations, αM showed less cell growth inhibition compared to those formulated in the form of nanoparticles (αM-Ch and αM-Ch/Cr).

The use of biopolymers or a combination of excipients derived from nature often has the advantage to prevent drug efflux reactions that can reduce drug levels in the cytoplasm of cells [52], in contrast to those without using a combination, in general, drugs will be considered as foreign substances, which in the regulation will be released by the cell defense system, causing ineffective drug use [53]. The impressive results are shown by the formula encapsulated with carrageenan polymer, which shows an increase in cytotoxicity of αM in inhibiting the growth of cancer cells. This is because carrageenan is known to have an influence on the regulation of cancer cells. Previous studies have shown that carrageenan can inhibit the migration of MDA-MB-231 breast cancer cells [54]. In addition, carrageenan has also been investigated for its anticancer activity in human cervical carcinoma cells (HeLa) and human umbilical vein endothelial cells (HUVEC); these studies show that carrageenan has activity in the specific cell cycle phase of human cervical carcinoma cells [55]. Carrageenan mechanism in inhibiting the growth of cancer cells has also been investigated, where carrageenan is able to induce the process of apoptosis through activating the pathways of caspase-3, caspase-8, and caspase-9; remodulation of the Bax:Bcl-2 ratio; and DNA damage [56]. This is probably what causes an increase in the cytotoxicity of αM encapsulated with kappa carrageenan polymer against MCF-7 breast cancer cell lines.

## 5. Conclusions

In summary, αM-Ch/Cr nanoparticles were successfully formulated and evaluated for their anticancer potential in MCF-7 breast cancer cells. αM-Ch/Cr nanoparticles showed increased solubility of αM-Ch/Cr, and the in vitro release test showed a sustained release pattern. In addition, the cytotoxicity study confirmed that αM-Ch/Cr αM has increased the cytotoxicity of αM in MCF-7 breast cancer cells. These results suggest that αM-Ch/Cr nanoparticles have the potential to improve the physicochemical properties and cytotoxicity effects of αM compounds as a candidate for breast cancer therapy agents.

## Figures and Tables

**Table 1:** αM polymeric nanoparticle formulation.

Formulation	F1	F2	F3
αM (mg)	20	20	20
Chitosan (mg)	200	200	200
Sodium tripolyphosphate (mg)	40	40	40
Kappa carrageenan (mg)	25	45	85

**Table 2:** The mean entrapment efficiency and drug loading of the nanoparticles.

Formulae	Entrapment Efficiency (%)	Drug Loading (%)
F1	98.74 ± 1.20	6.93 ± 0.87
F2	99.35 ± 1.10	6.51 ± 0.86
F3	98.59 ± 0.98	5.72 ± 0.60

**Table 3:** FTIR wavenumber and corresponding functional groups.

Material	Wavenumber (cm^−1^)		Functional Groups	Reference
	Result	Literature		
α-mangostin	34,081; 32,204	3260	O–H *stretch*	[37]
	29,875; 26,078; 29,317	2989; 2962; 2924	C–H *stretch*	
	16,338	1642	C=O	
	14,435	1454	C–C	
	11,782	1199	Ortho–OCH_3_ *stretch*	
	107,533	1076	C–O–C *stretch*	
Chitosan	350,954	347,868	O–H *stretch* dan N–H *stretch*	[38]
	289,424	292,413	C–H *stretch*	
	165,302	165,688	C=O	
	1597	157,105	N–H *bend*	
	141,963	142,253	C–H *bend*	
	137,816	137,816	C–N	
	115,731	115,731	C–O–C *stretch*	
	1079	102,518	C–O	
Sodium tripolyphosphate	121,132	1210	ν_as_ P=O	[39]
	115,731	1130	ν_s_ O–P=O	
	109,269	1090	ν_as_ PO_3_	
	88,823	888	ν_as_ P–O–P	
Kappa carrageenan	1262	1241	O=S=O	
	1069	1069	Glycosidic bond	
	929	922	3,6-anhydrogalactose	[40]
	846	847	Galactose-4-sulfate	

## Data Availability

Samples of the data are provided by the corresponding author on request.
